# The instrumented single leg stance test detects early balance impairment in people with multiple sclerosis

**DOI:** 10.3389/fneur.2023.1227374

**Published:** 2023-07-19

**Authors:** Pål Berg-Hansen, Stine Marit Moen, Thomas Dahl Klyve, Victor Gonzalez, Trine Margrethe Seeberg, Elisabeth Gulowsen Celius, Andreas Austeng, Frédéric Meyer

**Affiliations:** ^1^Department of Neurology, Oslo University Hospital, Oslo, Norway; ^2^MS Center Hakadal, Hakadal, Norway; ^3^SINTEF Digital, Smart Sensor and Micro Systems, Oslo, Norway; ^4^Institute of Clinical Medicine, University of Oslo, Oslo, Norway; ^5^Department of Informatics, University of Oslo, Oslo, Norway

**Keywords:** multiple sclerosis, single leg stance test, inertial measurement units, wearable sensors, biomechanics

## Abstract

Balance impairment is frequent in people with multiple sclerosis (pwMS) and affects risk of falls and quality of life. By using inertial measurement units (IMUs) on the Single Leg Stance Test (SLS) we aimed to discriminate healthy controls (HC) from pwMS and detect differences in balance endurance and quality. Thirdly, we wanted to test the correlation between instrumented SLS parameters and self-reported measures of gait and balance. Fifty-five pwMS with mild (EDSS<4) and moderate disability (EDSS≥4) and 20 HC performed the SLS with 3 IMUs placed on the feet and sacrum and filled the Twelve Item Multiple Sclerosis Walking Scale (MSWS-12) questionnaire. A linear mixed model was used to compare differences in the automated balance measures. Balance duration was significantly longer in HC compared to pwMS (*p* < 0.001) and between the two disability groups (*p* < 0.001). Instrumented measures identified that trunk stability (normalized mediolateral and antero-posterior center of mass stability) had the strongest association with disability (R^2^ marginal 0.30, *p* < 0.001) and correlated well with MSWS-12 (*R* = 0.650, *p* < 0.001). PwMS tended to overestimate own balance compared to measured balance duration. The use of both self-reported and objective assessments from IMUs can secure the follow-up of balance in pwMS.

## Introduction

Multiple sclerosis (MS) is a chronic neurological disease of the central nervous system, currently affecting 2.8 million people worldwide (1). Gait problems are among the most frequent complaints in people with MS (pwMS) (2) and constitutes of several components, including walking speed and balance. Balance impairment affects approximately 75% of pwMS during the disease course (3) and is one of the most debilitating symptoms (4). PwMS have a higher risk of falls than healthy peers (5), even in early phases of the disease (6, 7). Falls in pwMS are associated with a higher risk of serious injuries (8) as well as a change in behavior related to fear of falling (9). Trusting own balance is thus important for maintaining a good quality of life. Expanded Disability Status Scale (EDSS) (10) is the most frequently used disability measure in pwMS, but has major limitations, and does not specifically measure balance problems (11). A standard neurological examination includes qualitative tests of balance, such as Romberg’s test (12) and the tandem gait test (13). Balance tests used in studies and follow-up of pwMS, such as the Time Up and Go Test (TUG) (14) and Berg Balance Scale (15), are complex and time consuming. Single Leg Stance Test (SLS) is widely used in elderly (16) and in people with neurological disorders, such as Parkinson’s disease (17). A few studies including SLS have also been reported in pwMS (18–20).

Wearable technology is an evolving field in modern medicine. Inertia motion sensors (IMUs) have been used as a tool to evaluate qualitative patterns of gait in different study populations, including MS (21, 22) and offer a more objective measure than clinical evaluation of therapists. In a recent publication we confirmed differences in walking distance in the six-minute walk test (6MWT) by pwMS with low (EDSS<4) and moderate (EDSS ≥4) disability (23). Adding IMUs gave additional information about gait quality and identified differences in the effects of rehabilitation between pwMS with different disability levels. Balance has also been evaluated using different kinds of sensors in MS, i.e., stance with eyes open or closed at different surfaces, TUG, timed 25-foot walk test, 6MWT and with external perturbations (24). The instrumented 30-s chair stand test was shown to predict risk of falls in pwMS (25).

The underlying mechanisms leading to balance problems in pwMS are complex, involving the cortico-spinal tract, proprioceptive pathways, cerebellum, brainstem, the visual and the vestibular system (26). Anticipatory postural adjustments (APA) are important in keeping the center of mass stable prior to a body perturbation, such as preparing to stand on one leg (27). Studies of pwMS initiating walking identified a simplified APA pattern in early phases of MS compared to healthy controls (HC) (28). In a recent paper, instrumented SLS with three IMUs was able to discriminate between HC and three groups of people with parkinsonism, based on objective characterization of the APA phase (29).

Patient-reported outcome measures are more often included in clinical studies. The twelve item Multiple Sclerosis Walking Scale (MSWS-12) (30), a self-reported questionnaire on walking ability in MS, is shown to correlate with improvement after rehabilitation, especially in early phases of the disease (31). Question 5 (Q5) in the MSWS-12 questionnaire is a specific question about self-experienced balance.

The aim of this study was to examine whether SLS with IMUs could discriminate healthy controls from pwMS at different disability levels. We also wanted to examine which components of the test better explains the differences in balance endurance and quality. Thirdly, we wanted to test the correlation between MSWS-12 and SLS in general, and more specifically Q5, the question on self-reported balance. In addition we wanted to examine the potential effect of a short rehabilitation stay on automated balance measures.

## Materials and methods

### Participants

The participants in this study are partly overlapping with those included in our previous study on 6MWT where methods are described into more detail (23). Nine additional pwMS were tested since the first study with the same setup at the MS Center Hakadal. Thus 55 pwMS and 20 HC were available for inclusion. Due to technical issues with synchronization of the IMUs 41 pwMS and 15 HC were included in the present study. All participants signed a written consent form. Five of the pwMS were diagnosed as primary progressive MS and 36 with relapsing remitting MS. The pwMS were divided in a mild-disability group (EDSS <4) and a moderate-disability group (EDSS ≥4). None of the pwMS had changed disease modifying treatment or had any clinical relapses in the last month prior to inclusion. Thirteen of the patients were using fampridine that may improve walking range and balance in pwMS (32, 33). Clinical data for the pwMS was retrieved from the medical files and are shown in Table 1.

**Table 1:** Clinical and demographic data of people with multiple sclerosis (pwMS) and healthy controls (HC).

	HC (*n* = 15)	pwMS (*n* = 41)	Value of *p* (pwMS vs. HC)	EDSS<4 (*n* = 19)	EDSS> = 4 (*n* = 22)	Value of *p* (EDSS<4 vs. EDSS= > 4)
Age in years, mean (SD)	45.2 (13.3)	49.3 (8.8)	0.19	48.0 (11.10)	50.5 (6.3)	0.36
Female/male (% female)	12/3 (80.0)	26/15 (63.4)	**<0.001**	14/5 (73.7)	12/10 (54.5)	0.21
BMI, mean (SD)	25.2 (3.2)	27.8 (5.5)	0.10	30.1 (4.0)	25.66 (5.8)	**0.01**
EDSS, median (range)	–	4.0 (1–6)		2.5 (1–3.5)	4.5 (4–6)	**<0.001**
Years since diagnosis, median (range)	–	9.0 (0–30)		8.0 (0–30)	9.5 (0–21)	0.93

BMI, Body Mass Index; SD, Standard Deviation; EDSS, Expanded Disability Status Scale.Significant p values are marked with bold.

### Test procedure

The participants were tested under the same test conditions described previously (23). A neurological exam was performed prior to the testing with assessment of EDSS. The pwMS filled in the MSWS-12 questionnaire before the test procedure. The participants were instructed as follows: *Look straight ahead. Keep your hands on your hips. Lift your leg off the ground behind you without touching or resting your raised leg upon your other standing leg. Stay standing on one leg as long as you can.* Both legs were tested twice alternating between the two legs. Each trial was stopped if the participant was standing for 60 s on one leg.

### Equipment

Three IMUs (Physiolog 5® from GaitUp SA, Lausanne, Switzerland) containing a 3D accelerometer and a 3D gyroscope were placed on both feet and on the sacrum with an elastic band. The sampling rate of the accelerometer and gyroscope was set to 128 Hz with a range of +/− 8 g for the accelerometer and +/− 1,000 deg./s for the gyroscope.

### Analysis

The leg with the longest test duration was defined as the *least affected leg* independently of whether it was the persons strongest or dominant leg. The best performance of the two attempts for each leg was used in the calculations. The SLS test was subdivided in three sequences: *time-to-peak*, *peak-to-balance* and *balance duration* as defined from the paper by Bonora et al. (29). The time-to-peak consist of the duration between the start of the trunk medio-lateral motion to the time of maximal medio-lateral acceleration of the trunk (Tpeak). The peak-to-balance is the duration between Tpeak and the start of the balance phase (Tstart) when the foot is lifted. The balance duration is measured between the Tstart, and the time when the foot touches the ground again. The maximal medio-lateral acceleration (peak-acceleration) of the trunk during the APA phase was also reported, as well as the root-mean-square (RMS) values of the trunk mediolateral and antero-posterior acceleration during the balance phase. The RMS values were also divided by the duration of the balance phase to provide normalized values.

### Statistics

Normally distributed continuous variables were compared using independent samples *t*-test. Pearson’s Chi-square test was used to compare dichotomous variables. Mann–Whitney U test was used when comparing ordinal variables. Age and BMI for the HC and two groups of pwMS were compared with One-way ANOVA with Tukey *post hoc* analysis, and with Kruskall-Wallis test for gender distribution. A linear mixed model was used to compare duration of the different phases of the SLS test between HC and pwMS. The significance level was set at *p* < 0.05. Bonferroni corrections were performed to adjust for multiple calculations. For the R^2^ effect sizes values above 0.02 were considered small, 0.15 medium and 0.35 large (34). The analyzes were performed using the Jamovi Software (Jamovi project 2020, version 1.8.1.0).

## Results

The clinical and demographic data of the pwMS and HC are given in Table 1. There were no significant differences in age or gender distribution between the HC and pwMS, nor internally between the two disability groups. The mild disability group had a significantly higher Body Mass index (BMI) compared to the moderate disability group and HC (both *p* = 0.01). There was no significant difference in disease duration between the two disability groups. The mean MSWS-12 score for the pwMS was 52.2 (SD 22.7). It was 37.5 (19.6) for the EDSS<4 group and 65.0 (17.1) for the EDSS≥4 group (*p* < 0.001). For the MSWS-Q5, it was 3.0 (0.9), 2.4 (0.7) and 3.5 (0.8), respectively (p < 0.001 between the two disability groups).

**Figure 1:**
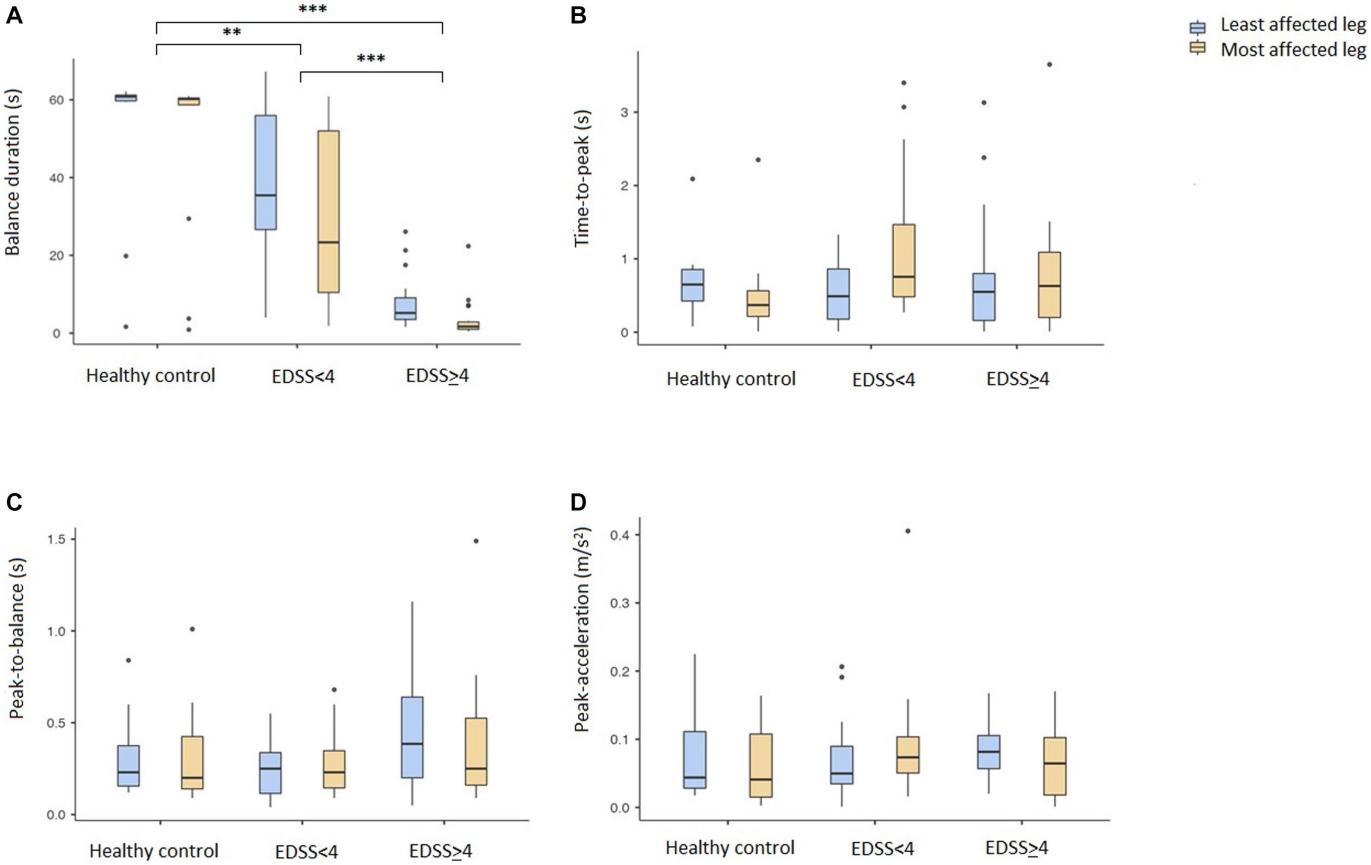
Duration of the three phases and peak-acceleration of the instrumented single leg stand test. Mean values with 95% confidence intervals are given for the HC and two MS disability groups (EDSS<4 and EDSS≥4). Least affected leg is marked with blue and most affected leg in yellow. **(A)** Balance duration, **(B)** Time-to-peak, **(C)** Peak-to-balance, **(D)** Peak acceleration. Lines indicate significant differences between groups and legs. *p < 0.05, **p < 0.01, ***p < 0.001. EDSS, Expanded Disability Status Scale.

**Figure 2:**
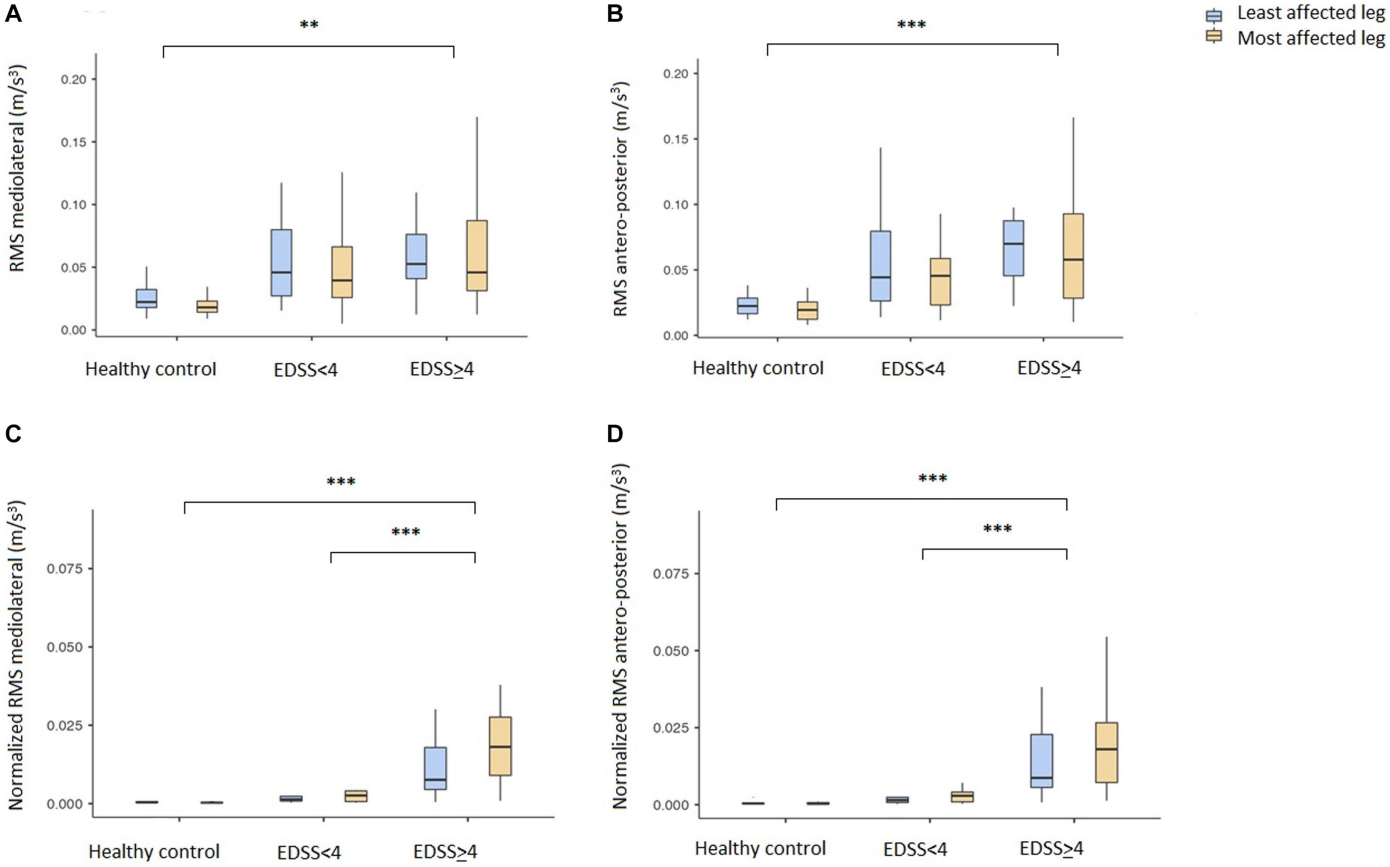
Center of mass stability measures derived from IMU on sacrum during the instrumented single leg stand test. Mean values with 95% confidence intervals are given for the healthy controls (HC) and two MS disability groups (EDSS<4 and EDSS≥4). Least affected leg is marked with blue and most affected leg in yellow. **(A)** Mediolateral center of mass stability (RMS mediolateral), **(B)** Antero-posterior center of mass stability (RMS antero-posterior), **(C)** Normalized mediolateral center of mass stability (Normalized RMS mediolateral), **(D)** Normalized antero-posterior center of mass stability (Normalized RMS antero-posterior). Lines indicate significant differences between groups and legs. *p < 0.05, **p < 0.01, ***p < 0.001. IMUs, Inertia measurement units; EDSS, Expanded Disability Status Scale; RMS, Root-mean-square.

The results derived from the sensor data during the instrumented SLS test are shown in Table 2 and Figures 1, 2. Data shown after adjusting for multiple testing. There were significant differences in balance duration between the HC and both disability groups respectively, and between the two pwMS groups (Figure 1A), with a large effect size (*R*^2^ = 0.618). It was also a significant difference between the two legs. There were no significant differences in time-to-peak (APA) or in peak-to-balance (time to stabilization) between the groups (Figures 1B,C). The peak-acceleration (Figure 1D) was neither different between the HC and pwMS groups. The parameters RMS mediolateral and RMS antero-posterior were significantly different only for HC compared to the moderate disability pwMS, but not between the two EDSS groups (Figures 2A,B). There were significant differences in the parameters normalized RMS mediolateral and normalized RMS antero-posterior between HC and EDSS≥4, and between the two pwMS group (Figures 2C,D). The parameter normalized RMS antero-posterior was also significantly different between the most and least affected leg.

**Table 2:** Duration of SLS phases and center of mass stability measures for HC and pwMS derived from IMUs.

Parameter	HC (*n* = 20)		EDSS<4 (*n* = 19)		EDSS≥4 (*n* = 22)		Effect size	Leg	Group	HC vs. EDSS<4	HC vs. EDSS≥4	EDSS<4 vs. EDSS≥4
	Least affected leg	Most affected leg	Least affected leg	Most affected leg	Least affected leg	Most affected leg	r2 marginal	*p*	*p*	*p*	*p*	*p*
Balance duration (s)	54.2 ± 18.0	50.3 ± 21.0	38.5± 8.9	28.6 ± 22.8	7.92 ± 6.52	3.48 ± 4.88	0.618	<0.001	<0.001	0.006	<0.001	<0.001
Time-to-peak (s)	0.66 ± 0.48	0.51 ± 0.56	0.56 ± 0.43	1.20 ± 1.06	0.73 ± 0.83	0.78 ± 0.83	0.032	0.307	0.331	–	–	–
Peak-to-balance (s)	1.38 ± 0.59	1.56 ± 0.57	1.29 ± 0.24	1.31 ± 0.39	1.19 ± 0.64	1.49 ± 0.60	0.045	0.051	0.525	–	–	–
Peak-acceleration (m/s^2^)	0.075 ± 0.064	0.061 ± 0.054	0.071 ± 0.064	0.099 ± 0.097	0.085 ± 0.045	0.066 ± 0.052	0.012	0.682	0.679	–	–	–
RMS mediolateral (m/s^3^)	0.029 ± 0.023	0.020 ± 0.009	0.059 ± 0.043	0.052 ± 0.037	0.076 ± 0.059	0.065 ± 0.048	0.184	0.106	0.002	0.084	0.002	0.695
RMS antero-posterior (m/s^3^)	0.025 ± 0.014	0.028 ± 0.030	0.055 ± 0.038	0.048 ± 0.036	0.073 ± 0.043	0.069 ± 0.050	0.197	0.604	<0.001	0.125	<0.001	0.288
Normalized RMS mediolateral (m/s^3^)	0.002 ± 0.004	0.002 ± 0.003	0.003 ± 0.004	0.004 ± 0.005	0.016 ± 0.019	0.023 ± 0.022	0.288	0.130	<0.001	1.000	<0.001	<0.001
Normalized RMS antero-posterior (m/s^3^)	0.001 ± 0.003	0.002 ± 0.007	0.002 ± 0.003	0.001 ± 0.003	0.014 + 0.013	0.025 ± 0.026	0.300	0.046	<0.001	1.000	<0.001	<0.001

SLS, Single Leg Stance Test; HC, healthy control; pwMS, People with MS; IMUs, Inertia measurement units, EDSS, Expanded Disability Status Scale; RMS, Root-mean-square. The table shows mean values ± standard deviations for the different phases and for the mediolateral and antero-posterior center of mass stability in the single leg stand test for HC and pwMS in the two disability groups. Data shown for the least and most affected leg, respectively, and was drawn from the sensor on sacrum. Statistics from the mixed model is shown in the six last columns with effect size and *p* values for the two legs, 3 groups and with post-hoc comparison between HC and the two pwMS disability groups.

**Figure 3:**
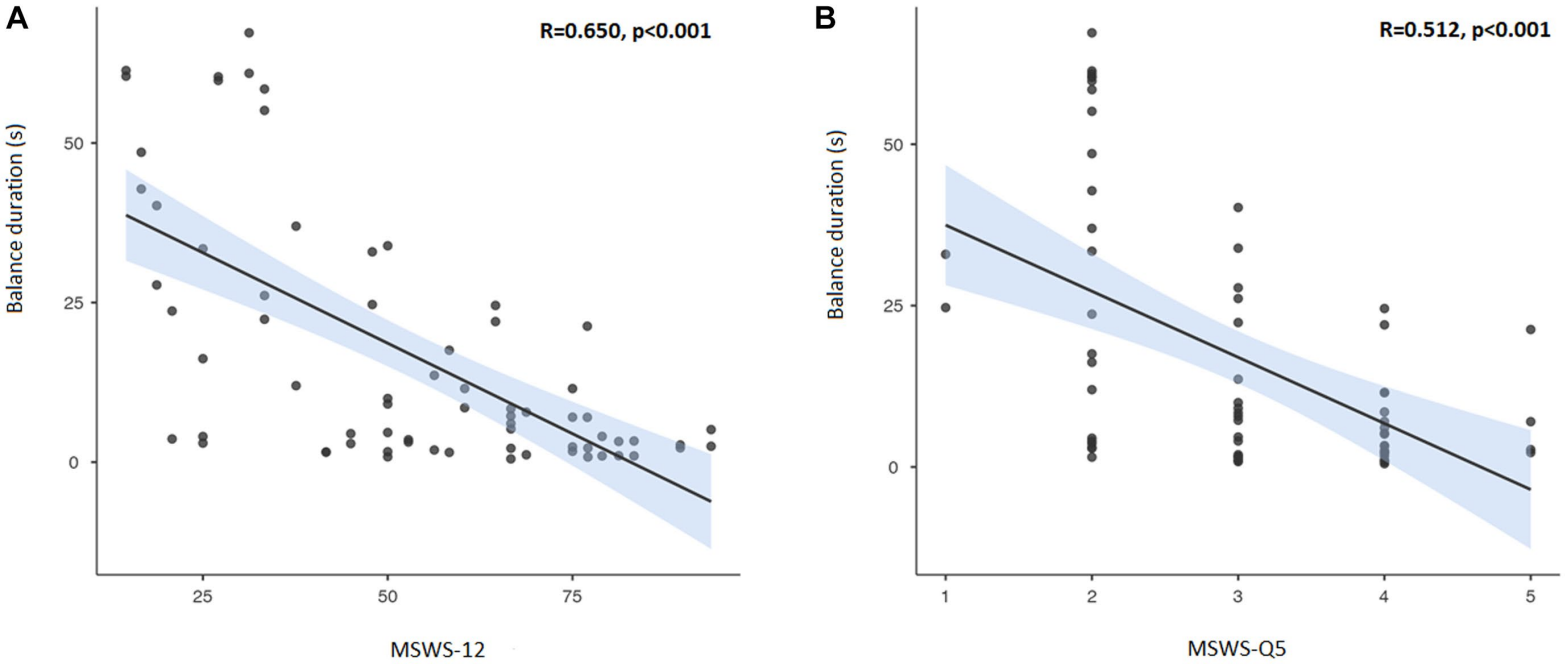
Correlation between MSWS-12 **(A)** and MSWS-Q5 **(B)** with balance duration of the single leg stand test. Correlation line in black with 95% confidence interval marked in blue. MSWS-12, Twelve Item Multiple Sclerosis Walking Scale; Q5, Question 5 in the MSWS-12.

The correlation between the balance duration of the SLS test and MSWS-12 total score (3A) and the MSWS-Q5 (3B) respectively, is shown in Figure 3. The correlation coefficients indicate a good correlation between self-reported walking ability and total duration of SLS test. There was a lower but still good correlation between MSWS-Q5 and balance duration.

**Figure 4:**
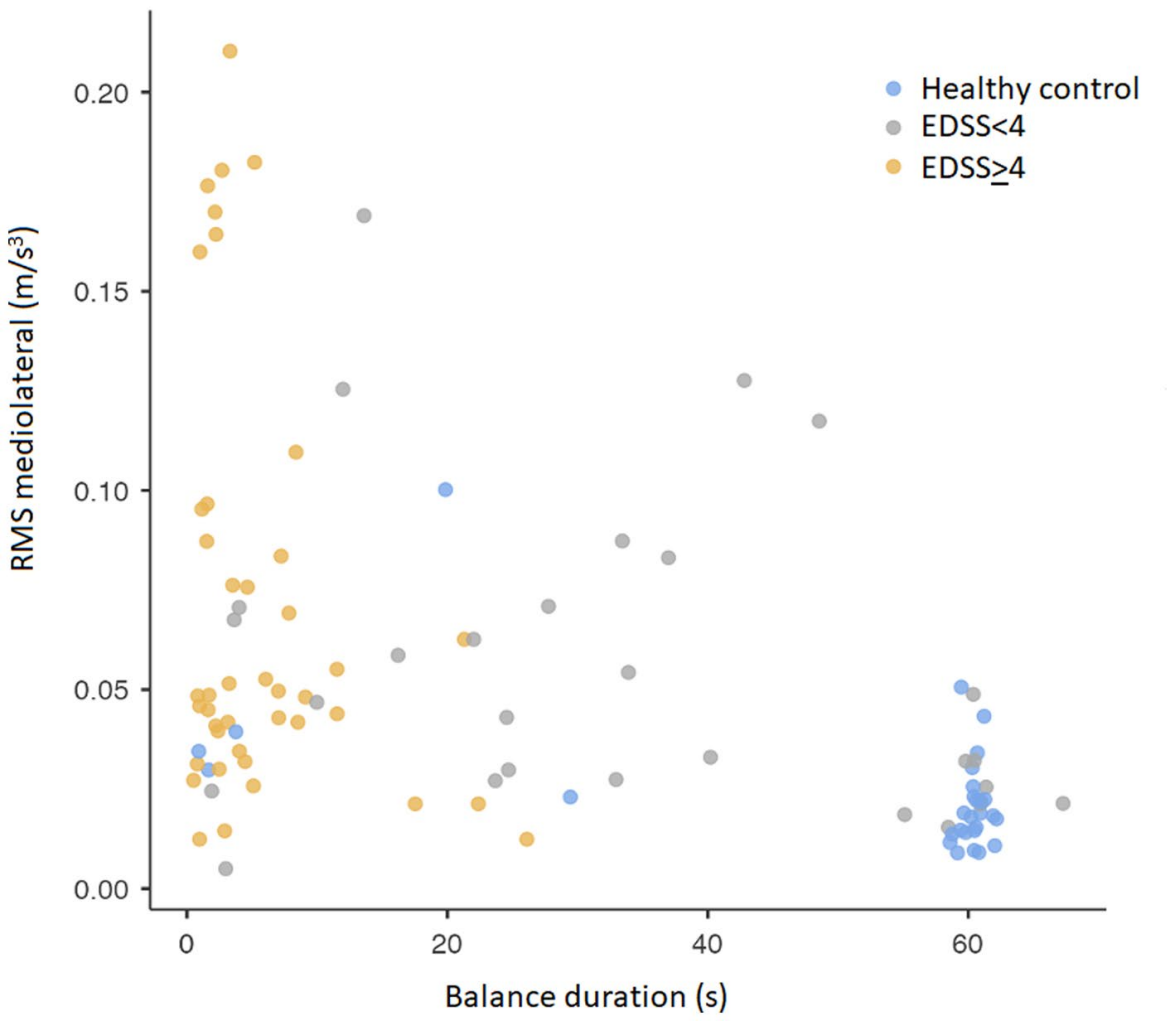
Correlation between balance duration and root-mean-square in the mediolateral direction of the sensor on sacrum. RMS, Root-mean-square.

The correlation between duration of the balance phase and RMS in the mediolateral direction for HC and the two pwMS disability groups is presented in Figure 4. Overall, there was a strong correlation between the two parameters (*R* = 0.798, *p* = 0.026). There was a significant difference between HC and both pwMS groups (both *p* < 0.001). As the figure illustrates, most HC stand long and stable on one leg. The EDSS<4 group stand shorter and are less stable, while most of the EDSS≥4 group stand less than 10 s. There are, however, a high variation in trunk stability among the most disabled patients.

As a quality control the correlation between the balance duration recorded by the IMUs and the “stop-watch” timed duration reported by the clinicians was excellent (*R* = 0.947, *p* < 0.001; Supplementary Figure S1).

Most of the pwMS performed a retest of the SLS test at the end of the rehabilitation stay after a median of 15 days. There were, however, no significant differences in the test and retest results in the mixed model (*p* = 0.485).

## Discussion

In this first study of the instrumented single leg stand test in pwMS, we detected significant differences in balance duration as well as overall stability between HC and pwMS. The strongest and only large effect size was found for balance duration, which differentiated HC from pwMS with high and low disability and also differentiated between the two pwMS groups. Thus, the test is sensitive to discriminate even pwMS with minimal disability from healthy individuals. We found a high correlation between the automated tests and the timed test results reported from the clinicians. It was also a significant difference between the results from the two legs. Neither the initial time to stabilization (time-to-peak and peak-to-balance) nor the peak-acceleration correlated with disability level in pwMS. However, the center of mass stability in both the mediolateral and antero-posterior direction was significantly different for HC compared to the most disabled pwMS. The normalized values could also differentiate between the two pwMS groups. For the parameter normalized RMS antero-posterior we found a different stability between the legs only for the pwMS with ESSS ≥4, which is in line with a more severe disability.

The mean balance duration for the HC in this study was slightly longer than the reported normative values for the age group 40–49 year (41.2 s) (16). For the most disabled pwMS, mean balance duration was below 10 s, which was identified as a clinically important stage of disease progression associated with poor postural stability in people with Parkinson’s disease (17). The balance duration for both pwMS groups were longer than reported in a previous paper of SLS test in MS (18), however EDSS scores were not presented in that publication. Of notice, the most disabled pwMS in our study stood considerably shorter on either leg than those defined as *fallers* in the recent publications on different balance tests in pwMS (19). The number of people with progressive forms of MS were too few to include in the calculations in our study, as was done by Soyuer et al. (20).

The setup with three IMUs used on the SLS was similar to that used in the study of people with parkinsonism (29). We also used the same definition of the different phases of the test. In accordance with the parkinsonism study, we did find a significantly shorter balance duration in all pwMS compared to HC. There were neither any difference in the other temporal parameters time-to-peak nor peak-to-balance between the groups. We found significant differences for the normalized RMS in both mediolateral and antero-posterior direction between HC and pwMS, in line with a less stable stand phase in all patients and similar to the findings in the aforementioned study on people with parkinsonism. However, we did not find a difference in peak-acceleration in contrast to the parkinsonism study. It is important to keep in mind that pwMS and people with parkinsonism show different clinical patterns, though they are both chronic neurological diseases. Also of notice, 13 of 69 people with parkinsonism (but no pwMS in the present study) were not able to lift the leg at all and were thus excluded from the calculations. In a recent publication on the instrumented Romberg test by Carpinella et al. (35) sway complexity and intensity were reduced in early stage pwMS. These findings suggest a less automated control of balance, indicating a higher need for attention and early rehabilitation.

As expected, the mean MSWS-12 scores as well as the MSWS-Q5 score were lower for pwMS with low versus high disability. There was also a strong correlation between self-reported walking ability and balance compared to the measured balance duration. Interestingly, a relatively high proportion of pwMS in our study tended to overestimate their own gait ability and balance, which highlights the need for objective balance assessments in clinical practice. An active use of both self-reported and objective assessments can aid an individualized approach to reduce the risk of accidental falls and injuries.

There was an inverse correlation between balance duration and truncal stability. However, the most disabled pwMS showed a wide spectrum of trunk stability, indicating that general strength might affect static balance endurance more than pure proprioception. However, a very short balance duration for these pwMS indicates that they never managed to reach a stable phase and thus no strict conclusions can be made. This is an important aspect in clinical management and further research is warranted.

We found no effect of a short rehabilitation stay on balance measures. We have previously shown that the intervention at a MS rehabilitation center including physiotherapy did affect gait endurance as measured by the 6-MWT in the studied group (23). Our study did not aim to compare different treatment strategies or evaluate therapeutic elements. There were differences in content, duration, and volume of rehabilitation in this *real-world* study with data retrieved during rehabilitation stays of 2–4 weeks. The pwMS was a heterogeneous group on rehabilitation stays with common denominator a focus on physical rehabilitation related to mobility or walking. There was not focus on especially tailored or task-specific balance training. This might indicate that one should focus more specifically on balance training if better balance is the main goal in individual MS follow-up and rehabilitation. It could also mean that it is harder to affect balance than endurance through training, in line with a small study showing no effect of balance in pwMS after a 10 week rehabilitation program (36). However, another group found an effect both on balance measures (SLS not included) and risk of falling after twice-weekly intervention during 7 weeks (37). It has also been reported that 6 weeks of visuo-proprioceptive training improves balance as measured by the SLS and reduces falls in pwMS (38). Thus, more specified content, intervention time and a more specific training programs might be necessary to evaluate and improve balance compared to gait endurance.

The strengths of the present study are the use of a standardized, validated set up including commercially available IMUs. There was also a relatively high number of participants in each group allowing for meaningful statistical calculations. There are also some possible weaknesses in the study. The groups were comparable in relation to age, gender and disease duration, but BMI was higher among the least disabled pwMS. However, we are less concerned that BMI would affect balance as much as gait endurance. If relevant, this would have driven the results in the direction of a falsely smaller difference between the disability groups. The use of fampridine for some pwMS could have affected balance but would also have driven the results in the same direction. There was a relatively high dropout rate due to syncronization issues, which was due to wrong setting of the IMUs. In general Physilog inertial sensors have good technical capability compared to reference systems in healthy and clinical settings (39, 40). There were, however, no systematic differences between the sensored and included participants in the study that might have affected the results. To improve clinical relevance, future studies could also include validated fall risk scales to test for correlation with the instrumented SLS measures.

## Conclusion

In this study of pwMS and healthy controls, we found that the total SLS duration correlates well with disability level. Data recorded from IMUs revealed that trunk stability in the balance phase was the best parameter in discriminating pwMS from HC and between different disability groups. Recordings from the initial anticipatory postural adjustment phases do not discriminate on a group level but can give useful information on an individual level. The less disabled group showed the widest range of balance impairment and having a detailed analysis of the patients could potentially guide an individualized treatment. Self-reported data from the MSWS-12 including question 5 on balance correlates well with balance duration, but pwMS tend to overestimate own balance. An active use of both self-reported and such objective assessments from IMUs can improve and secure an individualized approach and follow-up of balance in pwMS.

## Data availability statement

The raw data supporting the conclusions of this article will be made available by the authors, without undue reservation.

## Ethics statement

The studies involving human participants were reviewed and approved by the local Data Protection Officer (DPO) at Oslo University Hospital (OUS) and MS Center Hakadal (MSSH). The patients/participants provided their written informed consent to participate in this study.

## Author contributions

PB-H, SMM, TMS, and FM designed the work. PB-H and TDK performed data acquisition. PB-H and FM analyzed data. PB-H, SMM, EGC, VG, AA, and FM contributed to the interpretation of the data. All the authors participated in the critical revision process and approved the manuscript.

## Funding

The project received support from the Norwegian research council (project number 270791). The open access publication fees were coved by the University of Oslo.

## Acknowledgments

The present study is part of a large research project AutoActive: Tools and Methods for Autonomous Analysis of Human Activities from Wearable Device Sensor Data and unites a multidisciplinary research team with partners from MS Center Hakadal, Norwegian University of Science and Technology, Olympiatoppen, OUS, SINTEF and University of Oslo.

## Conflict of interest

The authors declare that the research was conducted in the absence of any commercial or financial relationships that could be construed as a potential conflict of interest.

## Publisher’s note

All claims expressed in this article are solely those of the authors and do not necessarily represent those of their affiliated organizations, or those of the publisher, the editors and the reviewers. Any product that may be evaluated in this article, or claim that may be made by its manufacturer, is not guaranteed or endorsed by the publisher.
