# Supplementary figures and images for: The instrumented single leg stance test detects early balance impairment in people with multiple sclerosis
Source: Front Neurol. 2023 Jul 19;14:1227374. doi: 10.3389/fneur.2023.1227374 (PMC10394643; doi:10.3389/fneur.2023.1227374)

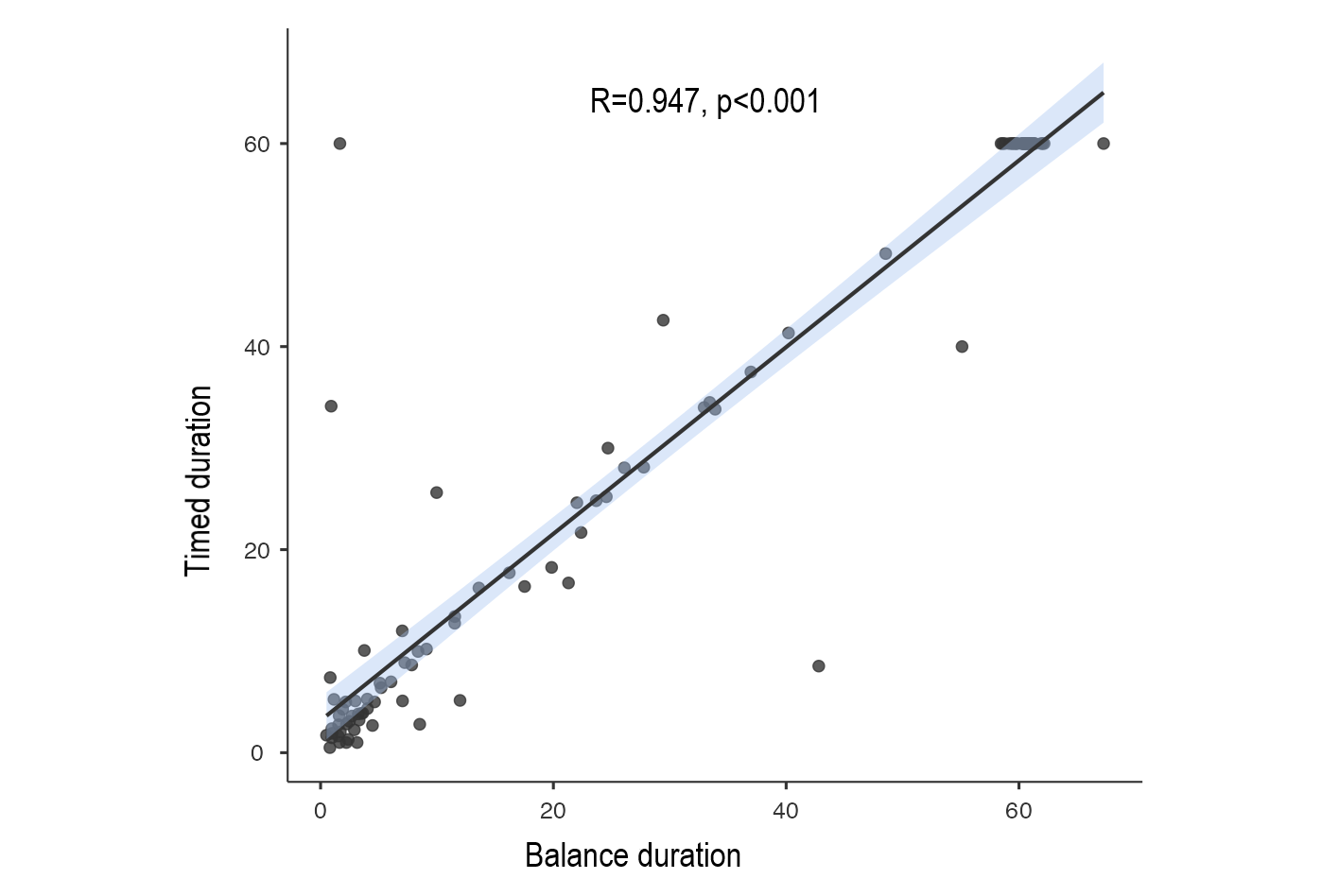

Supplement: Supplementary file 1 [file Image_1.TIFF]
